# Colonization of islands in the Mona Passage by endemic dwarf geckoes (genus *Sphaerodactylus*) reconstructed with mitochondrial phylogeny

**DOI:** 10.1002/ece3.770

**Published:** 2013-10-16

**Authors:** Alondra M Díaz-Lameiro, Taras K Oleksyk, Fernando J Bird-Picó, Juan Carlos Martínez-Cruzado

**Affiliations:** Department of Biology, University of Puerto Rico at MayagüezCall Box 9000, Mayagüez, Puerto Rico, 00681-9000

**Keywords:** Caribbean, Desecheo, island biogeography, Mona, mtDNA, *Sphaerodactylus.*

## Abstract

Little is known about the natural history of the *Sphaerodactylus* species endemic to the three islands located in the Mona Passage separating the Greater Antillean islands of Hispaniola and Puerto Rico. In this study, parts of two mitochondrial genes, 16S rRNA and 12S rRNA, were sequenced to determine the relationships between the sphaerodactylids that live in the Mona Passage and other Caribbean species from the same genus. While the main goal was to identify the biogeographical origin of these species, we also identified a genetically distinct type of dwarf gecko that warrants future evaluation as a possible new species. According to the reconstructed phylogenies, we propose a stepwise model of colonization wherein *S. nicholsi* from southwestern Puerto Rico or a very close ancestor gave rise through a founder event to *Sphaerodactylus monensis* on Mona Island. In a similar fashion, *S. monensis* or a very close ancestor on Mona Island gave rise to *S. levinsi* on Desecheo Island. This study also suggests that the most recent common ancestor between the species from the islands in the Mona Passage and Puerto Rico existed approximately 3 MYA.

## Introduction

**Figure 1:**
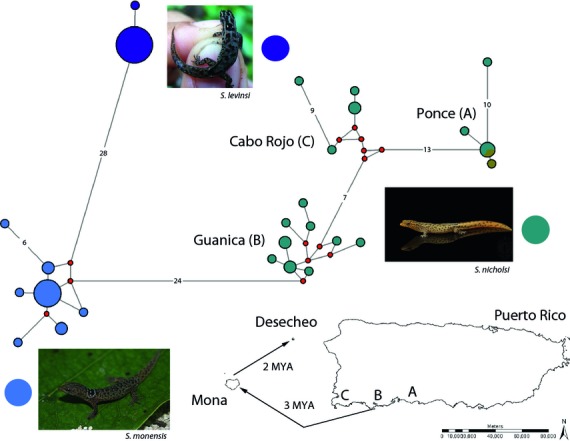
Median joining network of 16S and 12S rRNA concatenated sequences of four *Sphaerodactylus* species from southwestern Puerto Rico, Mona and Desecheo. Haplotypes are represented by circles, the size of which are proportional to their haplotype frequency. Colors identify species as indicated by photos of three of them. The fourth, *S. townsendi* of Ponce, is represented in olive. Hypothetical ancestors are represented by red diamonds. Distances exceeding three mutations separating haplotypes and hypothetical ancestors are indicated in the corresponding branches. Map of Puerto Rico illustrates the location of the three populations of *S. nicholsi*. Divergence time is in millions of years. Photo credit: *S. levinsi*, W. Falcon; *S. monensis*, J. Burgess; *S. nicholsi*, A. Gilpin.

The West Indies are a unique living evolutionary laboratory where islands are sufficiently isolated to sustain endemic forms, but close enough for the colonists to develop a dynamic interaction with the surrounding continental regions (Ricklefs and Bermingham 2008). Mona, Monito, and Desecheo are three islands located in the Mona Passage, between Puerto Rico and Hispaniola (Fig. 1). It is believed that the three islands represent independent biogeographical units (Heatwole and MacKenzie 1967). Desecheo lies on its own bank and has never have been connected to Puerto Rico (Thomas 1999). Mona and Monito, although close in proximity, are separated by a 275-m deep trench (Kaye 1959). During the glacial maxima, sea levels were much lower and the distance was even shorter (Heatwole and MacKenzie 1967), but the islands are unlikely to have ever been connected to either Hispaniola, Puerto Rico, or to each other (Kaye 1959; Heatwole and MacKenzie 1967).

Local species diversity on these islands is mostly a result of successful colonization events by overwater dispersal followed by allopatric divergence (Pregill 1981; Losos et al. 1997; Savolainen et al. 2006). Albumin evolution data support overwater dispersal as a primary mechanism of colonization in the West Indies (Hedges et al. 1992). Immunological distances measured between species from Hispaniola and Puerto Rico postdate the separation of those islands (Oligocene or early Miocene, 20–30 Ma), indicating that dispersal has occurred between the islands during the last 20 myr (Hedges et al. 1992).

The genus *Sphaerodactylus*, better known as dwarf geckos, consists of small lizards confined to the Neotropics. The tiny representatives of this genus (20–35 mm) can be found in leaf litter, under rocks, logs, or trash (Rivero 1998). *Sphaerodactylus* accounts for more than 80 species in the West Indies and up to 100 species in the Caribbean (Schwartz and Henderson 1991), where the genus reaches its greatest diversity (Gamble et al. 2008a). The diversification of dwarf geckos coincided with the period of increased connectivity of the Caribbean to South America at the beginning of the Oligocene (38–22 Ma) (Crawford and Smith 2005), when dwarf geckos gradually inhabited the dry forests of the Caribbean islands. Morphological forms of *Sphaerodactylus* are diverse, and it is believed that almost every island has developed a specific group (Grant 1931). Nine species of this genus are known from Puerto Rico, six of them on the main island, one on Desecheo, one on Mona, and one on the Monito Island next to Mona (Rivero 1998).

Geckos have two main characteristics that make them amenable to overwater dispersal: (1) They have eggs that are resistant to the effects of dehydration and temporary immersion in sea water (Brown and Alcala 1957), and (2) a special adhesive area at the tips of geckoes' digits allows them to hold on securely to flotsams (Russell 2002; Vanhooydonck et al. 2005). On the other hand, their small body size, relatively large surface area/volume ratio, and diurnal activity make them vulnerable to thermal stress and dehydration (Lopez-Ortiz and Lewis 2004).

This study focuses on the biogeographical history of two dwarf gecko species *Sphaerodactylus monensis* and *S. levinsi*. The former is a species endemic to Mona Island (Rivero 1998) (long. 67°51-57′ W, lat. 18°3-8′ N), located in the Mona Passage between Puerto Rico and Hispaniola. Mona Island consists of a tectonically uplifted carbonate plateau (Frank et al. 1998). With an area of 55.2 km^2^ and approximately 34 km of shore line (Woodbury et al. 1977), Mona is the biggest of the three islands in the Mona Passage. Its semi-arid climate is similar in rainfall, temperature, and the resulting vegetative growth to the Guánica area that supports a xeric forest habitat in the south coast of Puerto Rico (Woodbury et al. 1977; Heatwole et al. 1981). *S. levinsi* can be found under rocks and dead wood on the perimeters of dry gullies in forested areas of Desecheo Island (Meier and Noble 1990), a mountainous islet of 1.22 km^2^ and almost 4.8 km of shore line, located 21 km from Rincón on the western coast of Puerto Rico and 50 km north from Mona Island. The island is composed largely of deformed, fragmented volcanic rocks of early tertiary origin (Woodbury et al. 1971), calcareous mudstone, and sandstone (Seiders et al. 1972). Like Mona, Desecheo supports a xeric type forest (Heatwole et al. 1981).

When *S. levinsi* and *S. monensis* are compared morphologically with a number of geographically proximate species from the West Indies including *S. micropithecus* from Monito, *S. savagei* from Hispaniola, and *S. mariguanae* from the Bahamas, they group most closely with species of the Puerto Rico Bank, but are morphologically distinct from them (Schwartz 1977). Taxa from Mona Island usually show similar biogeographical patterns. Most vascular plants, butterflies, and lizards are believed to have colonized Mona Island from Puerto Rico, to the east, and not from the closer and larger potential donor source, Hispaniola, to the west (Woodbury et al. 1977; Smith et al. 1994; Rodríguez-Robles et al. 2007). This may be a consequence of the direction of the prevailing winds and currents: Puerto Rico is generally upwind and upstream from Mona, contrary to Hispaniola (Heatwole and MacKenzie 1967; Griffin et al. 2001). However, this pattern has several notable exceptions, as is the case of the Mona iguana *(Cyclura cornuta stejnegeri*), a subspecies of the *Cyclura cornuta* from the Dominican Republic (Rivero 1998; Malone and Davis 2004). From morphological analyses, Thomas and Schwartz (1966) concluded that *S. monensis* speciated by allopatry from an organism called *proto-macrolepis* that lived on the Puerto Rico Bank (Puerto Rico, Vieques, Culebra, and the Virgin Islands, excluding Saint Croix). Similarly, two hypothetical routes of *Sphaerodactylus* dispersion are proposed in this study: eastward from Hispaniola, similar to that of the Mona iguana, and westward from Puerto Rico, as in the majority of other reported dispersals.

To determine local patterns of biogeographical phylogeny of the endemic *Sphaerodactylus* species around the Mona Passage, we analyzed the 12S ribosomal RNA (12S rRNA) and 16S ribosomal RNA (16S rRNA) regions of the mitochondrial DNA (mtDNA) across different species. We constructed phylogenetic trees and haplotype networks to test the two biogeographical hypotheses and infer the dispersion patterns of the ancestor species to colonize the islands on the Mona Passage. In addition, divergence times between species were inferred to help align evolutionary events with the geological scale. Our findings add to the general knowledge of the biogeography of the Caribbean strengthening the general pattern of westward colonizations and can help guide future conservation efforts for the preservation of *S. monensis* and the rare and endangered *S. levinsi*. As little is known about the ecology of these two species, finding their phylogenetically closest relative can give some information on the ecology of the species according to the phylogenetic niche conservatism theory that predicts that closely related species occupy similar niches, while distant relatives are more ecologically dissimilar (Wiens 2004; Losos 2008). Furthermore, because of the apparent low terrestrial mobility of dwarf geckos, they may be one of the genera best suited for studies that aim to understand the relative importance in the colonization of these islands of flotsams originating and ending in different geographic regions through evolutionary time.

## Materials and Methods

### Sampling

A total of 113 samples of *Sphaerodactylus* species were collected in various sites on both sides of the Mona Passage and on two adjacent islands (Desecheo and Mona) in the Mona Passage (Table 1). Locations with suitable habitat (leaf litter, fallen debris, rocks, etc.) were targeted for the sampling efforts. Each individual caught was processed according to the IACUC permit (#2010112201) and, after proper identification and tail amputation, released at the collection site. Tail tissue was stored on ice or 75% alcohol, transported to the laboratory, and stored at −80°C until processed. Seven of the nine known *Sphaerodactylus* species from Puerto Rico were sampled. DNA sequences from additional samples were downloaded from the Nucleotide database of the National Center for Biotechnology Information (NCBI) website (http://www.ncbi.nlm.nih.gov) (Table S1).

**Table 1:** Collected samples, their localities, and sequences obtained

Island	Species	Location^3^,^4^	Samples	12S rRNA	16S rRNA
Puerto Rico	*S. macrolepis ateles*	Cabo Rojo	2	1	2
		Mayagüez	3	3	3
	*S. macrolepis guarionex*	Rincón	2	2	2
		Aguada	1	1	1
		Camuy^1^	1	1	1
	*S. nicholsi*	Cabo Rojo	7	7	7
		Guánica	6	6	6
		Ponce	19	4	4
	*S. roosevelti*	Guánica^1^	1	1	0
	*S. townsendi*	Ponce	2	2	2
	*S. klauberi*	Orocovis	12	11	12
	*Sphaerodactylus* spp.^2^	Rincón	10	10	10
	*Hemidactylus* spp.	Rincón	1	1	1
		Mayagüez	1	1	1
Desecheo	*S. levinsi*	Long Valley	18	18	18
		West Valley	2	2	2
Mona	*S. monensis*	Playa Mujeres	5	5	5
		Faro	6	6	6
		Bajura Los Cerezos	3	2	3
		Bajura Empalme	5	5	5
Hispaniola	*S. streptophorus*	Pedernales^3^	1	1	1
	*S. clenchi*	Las Terrenas^3^	1	0	0
		Samaná^4^	1	1	0
	*S. difficilis*	Hermanas Mirabal^4^	1	1	0
	*S. savagei*	La Altagracia^4^	1	1	0
		El Macao^3^	1	1	0

1 Shared by Juan Daza and Tony Gamble.

2 Samples could not be identified using the dichotomous key (Rivero 1998), and the name *Sphaerodactylus* spp. was assigned.

3 Samples from Hispaniola were originally collected by Daniel Scantlebury and acquired by a loan from Dr Richard E. Glor's Laboratory at the University of Rochester.

4 Sequence data were kindly shared by Dr Blair Hedges.

### Molecular methods

Total genomic DNA was extracted from tail tissue samples using the Tissue Mini Kit (Qiagen Inc., Valencia, CA, USA). Afterward, ethanol precipitation was performed to eliminate any residual ethanol from the extraction. Using total cellular DNA as a template, 12S rRNA (525 bp) and 16S rRNA (512 bp) fragments of the mtDNA were amplified through polymerase chain reactions (PCRs). The reactions were performed using the following primers: for 12S rRNA, primers Sph12S-F (5'AACTAGGATTAGATACCCTACTATGC3'), and Sph12S-R (5'TGCACTTTCCAGTACACTTACCA3') that were designed from information obtained in GenBank; for 16S rRNA, primers 16S-F (5'CTAACCGTGCAAAGGTAGCGTAATCAC3') (Gamble et al. 2008b), and 16d (5'CTCCGGTCTGAACTCAGATCACGTAG3') (Reeder 1995). PCRs were performed in 25 μL volumes consisting of 2 μL template DNA, 0.3 μL of GoTaq® Flexi DNA Polymerase (5 U/μL) (Promega Corporation, Madison, WI, USA), 1.3 μL of each primer (20 μmol/L), 2.5 μL 5X Colorless GoTaq® Flexi Buffer (Promega), 2 μL MgCl_2_ (25 mmol/L), 4 μL dNTP (2.5 mmol/L) (Bio-Rad Laboratories, Hercules, CA, USA), 1 μL bovine serum albumin (BSA) at 10 mg/mL, and 10.6 μL of ddH2O. The DNA was denatured at 94°C for 2.5 min and subjected to 35 cycles of amplification that consisted of one denaturing step at 94°C for 20 sec, one annealing step at 59.5°C (for 16S rRNA) or 53.2°C (for 12S rRNA) for 40 sec, and one extension step at 72°C for 1 min. The 35 cycles of amplification were followed by a final 5 min elongation step at 72°C. For every PCR, an electrophoresis was carried out with 3 μL of PCR product on a 2% agarose gel and later stained with ethidium bromide to verify product band size and successful amplification. All PCR products were purified using the High Pure PCR Product Purification Kit (Roche Applied Science, Indianapolis, IN, USA). Afterward, another ethanol precipitation was performed to eliminate any residues of ethanol in the amplicons. Approximately half of the sequenced reactions were carried out at the University of Puerto Rico at Mayagüez facilities using the Big Dye 3.1 Cycle Sequencing Terminator Kit (Applied Biosystems, Foster City, CA, USA) and an ABI 3130 Genetic Analyzer (Applied Biosystems) as described by the manufacturer. The remainder was outsourced to the Nevada Genomics Center of the University of Nevada at Reno. The same primers described before were used for sequencing both DNA fragments.

### Data analyses

Each sequenced DNA was edited using Chromas 1.62 software (Technelysium, Helensvale, Qld, Australia) and Omiga 2.0 software (Oxford Molecular, Summertown, Oxford, UK). The edited matrix of good quality sequence was 365 and 378 bp long for the 12S and 16S RNA ribosomal fragments, respectively. All the alignments were pursued using the Phylemon 2 website (http://phylemon.bioinfo.cipf.es/utilities.html) with the Muscle 3.7 alignment algorithm (Edgar 2004) and its default parameters. To study phylogenetic relationships, phylogenetic trees were built through Bayesian inference and maximum likelihood methods using MrBayes 3 (Ronquist and Huelsenbeck 2003) and PhyML 3.0 (Guindon et al. 2010), respectively. Bayesian inference trees were constructed using the General Time Reversible Model with a proportion of invariable sites and a gamma-shaped distribution of rates across the sites with one million repetitions. Maximum likelihood trees were constructed under the K80 model and bootstrapped with 1000 repetitions. The Subtree Pruning and Regrafting algorithm was used for tree search. DnaSP 5.10 (Librado and Rozas 2009) was used to convert sequence files into rdf files to facilitate the construction of networks. This software was also used to calculate haplotype diversity (π) and perform the Tajima's D test. Median-joining mutational networks (Bandelt et al. 1999) were constructed using Network 4.516 software (Flexus-Engineering, Clare, Suffolk, UK). To estimate TMRCA between each pair of species, we obtained consensus sequences for each species through Omiga 2.0 and applied to each species pair a Bayesian relaxed clock method available in BEAST version 1.4.8 (Drummond and Rambaut 2007). Divergence dates were calculated using the Hasegawa, Kishino, and Yano (HKY) model with four gamma categories and the length of the chain set to 10 million as instructed by the package. The rate of 1.5% divergence per million years (0.75% per lineage per million years) was assumed for mtDNA and used for inferring the divergence times. This rate is the one used at various nodes dated using multiple geological calibration points and correlated genetic distances with other *Sphaerodactylus* species (Thorpe et al. 2008; Surget-Groba and Thorpe 2013). TRACER version 1.4 (Rambaut and Drummond 2007) was used to obtain the mean divergence time of TMRCA and its standard deviation (Table 2).

**Table 2:** Mean divergence time estimates with standard deviations in millions of years calculated with a 1.5% divergence per million years rate

	SMO	SL	SMA	SMG	SN	ST	SK	SPP
SMO	0	2.6 ± 5.5	11.1 ± 2.5	11.2 ± 2.3	3.4 ± 6.9	3.4 ± 9.2	5.2 ± 1.0	5.2 ± 1.5
SL		0	11.1 ± 2.9	11.2 ± 3.0	3.4 ± 6.3	3.5 ± 7.4	5.2 ± 1.3	5.2 ± 9.9
SMA			0	0.7 ± 3.0	11.1 ± 1.9	11.2 ± 2.9	11.1 ± 1.9	11.2 ± 2.6
SMG				0	11.6 ± 2.0	11.2 ± 3.0	11.1 ± 2.2	11.1 ± 2.6
SN					0	1.2 ± 4.2	5.2 ± 1.1	5.2 ± 9.9
ST						0	5.2 ± 1.0	5.2 ± 9.0
SK							0	3.7 ± 8.1
SPP								0

SMO, *S. monensis*; SL, *S. levinsi*; SMA, *S. macrolepis ateles*; SMG, *S. macrolepis guarionex*, SN, *S. nicholsi*; ST, *S. townsendi*; SK, *S. klauberi*; SPP, *Sphaerodactylus* spp.

## Results

### Haplotype networks

Individual networks were constructed for each gene sequenced with all the samples that were collected by the authors (Table 1) and others downloaded from GenBank (Table S1). Networks are phylogenetic representations that map genetic relationships between closely related organisms without a specific root or known ancestor. Both networks (16S rRNA and 12S rRNA) formed various distinct clusters consisting of Hispaniolan, Puerto Rican only, or Puerto Rican and Mona Passage species (Fig. S1). Both networks indicated that *S. monensis* and *S. levinsi* were connected to the species in Puerto Rico by shorter branches (8 mutations at 16S rRNA and 12 mutations at 12S rRNA from *S. monensis* to *S. nicholsi*) than to those in Hispaniola (>40 mutations from *S. monensis* to any of the Hispaniola *Sphaerodactylus*). However, the networks differed in the sequential speciation of the Mona Passage species and *S. nicholsi* from the bulk of species in Puerto Rico and Hispaniola. If the roots of the networks are placed within the bulk of species in Puerto Rico and Hispaniola, the 16S network shows *S. levinsi* diverging from *S. monensis*, which in turn diverged from *S. nicholsi*. The 12S rRNA network shifts *S. nicholsi* from being ancestral to the Mona Passage species to being derived from them. To construct a more robust median-joining haplotype network, the 12S rRNA and 16S rRNA sequences were concatenated, forming a 722-bp-long sequence for each sample, (Fig. S2). This haplotype network only contains individuals sampled in this study in Puerto Rico and is consistent with the 16S rRNA haplotype network. In this analysis *S. macrolepis*, whose cluster includes two subspecies, *S. macrolepis ateles* and *S. macrolepis guarionex*, shows the highest haplotype diversity (π = 0.066) followed by *S. nicholsi* (π = 0.022). Species that show a low haplotype diversity, such as *S. klauberi* (π = 0.0019) and *Sphaerodactylus* spp. (π = 0), may be representing younger populations. Species that show low diversity and starlike haplotype distributions like *S. monensis* (π = 0.0034) and *S. levinsi* (π = 0.0004) may have gone through recent population expansions that produce negative values of Tajima's D (*S. monensis* = −1.38, *S. levinsi* = −1.72). However, neither Tajima's D value was statistically significantly different from zero. It can be observed that *S. nicholsi* is the most closely related species to *S. monensis* and *S. levinsi* (*S* = 0.12), in comparison with other Puerto Rico species represented in the network. *S. townsendi* shows two very closely related haplotypes, one of which is shared with *S. nicholsi*. This haplotype is among the *S. nicholsi* haplotypes least related to the Mona Passage species. The network also shows that *Sphaerodactylus* spp. and *S. klauberi* are sister species, sharing a hypothetical ancestor, but having a distinct differentiation between each other. These sister species are more closely related to *S. nicholsi* than *S. macrolepis* from northwestern Puerto Rico.

Because *S. macrolepis*, *S. klauberi,* and *Sphaerodactylus* spp. are very distant from the rest of the Mona Passage samples, they were removed from the analysis and the network was reconstructed only with *S. nicholsi, S. townsendi, S. monensis,* and *S. levinsi* samples to visualize the relationships between the populations in the Mona Passage (Fig. 1). *S. monensis* and *S. levinsi* form clusters distinct from one another by a large number of mutations. In general, the results are consistent with a stepping-stone dispersion from island to island. First, as expected for sequential founder events, a clear gradual decrease in diversity can be observed from the original to the derived species. Thus, haplotype diversity is highest in *S. nicholsi* (*π* = 0.022), lower in *S. monensis* (*π* = 0.0034), and lowest in *S. levinsi* (*π* = 0.0004). Second, an unresolved reticulation allows for *S. levinsi* and *S. monensis* to share an ancestral haplotype that is only one mutational step away from the founder *S. monensis* haplotype but 28 mutational steps from the *S. levinsi* founder. This suggests that *S. monensis* derived from *S. nicholsi* and that it may have given rise to *S. levinsi*.

Another important observation is that all *S. nicholsi* and *S. townsendi* haplotypes in Puerto Rico form distinct clusters according to their collection location (Fig. 1). The *S. nicholsi* in the Guánica area are closer to the species from the Mona Passage than the *S. nicholsi* from any other region, suggesting that the putative flotsam that gave rise to the Mona Passage species originated in the Guánica area.

**Figure 2:**
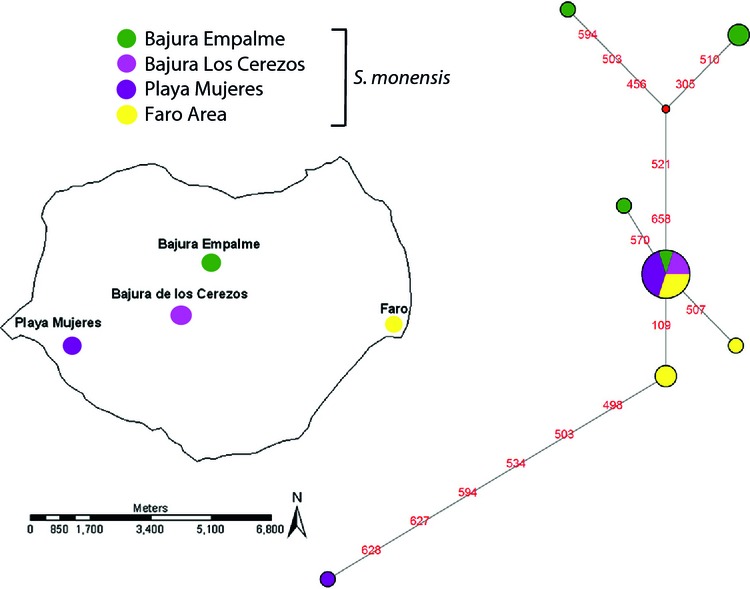
Median joining network of 16S and 12S rRNA concatenated sequences of *S. monensis* from Mona Island. Haplotypes are represented by circles, the size of which are proportional to their haplotype frequency. Colors identify collection sites. Hypothetical ancestors are represented by red diamonds. Mutations separating haplotypes are indicated by site number, where sites #1 to #345 correspond to the region encompassing from 591 to 956 in the 12S rRNA gene of *Gekko gecko* (Accesion # HM370130), and #346 to #696 correspond to the region of the 16S rRNA gene of *Gekko gecko* covering positions from 2105 to 2482.

Interestingly, the distribution of haplotypes from Mona reflects the geography of this island (Fig. 2). Among the seven haplotypes found, the most common was shared by ten individuals distributed across all collection sites. On the other hand, three haplotypes were exclusive to Bajura Empalme, two to the El Faro area, and one to Playa Mujeres. The starlike haplotype distribution suggests a rapid population expansion following colonization. Because of its frequency and dispersed distribution, the common central haplotype may represent the founder haplotype of Mona Island. The existence of local haplotypes suggests that gene flow across the island for this species is limited, and together with the clustering of *S. nicholsi* haplotypes according to collection sites, suggests that limited gene flow may be a characteristic of dwarf gecko populations.

### Phylogenetic trees

Phylogenetic trees of the 12S rRNA and 16S rRNA sequences were constructed using different sets of species downloaded from GenBank (Table S1) and two different algorithms (Bayesian inference and maximum likelihood). In general, the 12S rRNA tree (Fig. S3) had stronger bootstrap values than the 16S rRNA tree (Fig. S4). The 12S rRNA tree clearly divides dwarf geckoes into two groups: the ingroup, consisting of all the samples from Puerto Rico and Hispaniola, and the outgroup, which includes the species from the Lesser Antilles. The ingroup itself is divided into two separate clades. The first clade (Fig. S3; top) is represented by species from Puerto Rico, Mona, and Desecheo diverging from the only Hispaniolan species in the clade, *S. streptophorus*. Within the clade, *S. nicholsi* forms a subclade together with the Mona Passage species*, S. monensis* and *S. levinsi*. Another species from Puerto Rico, *S. klauberi*, is found ancestral to *S. nicholsi* and the Mona Passage species, and *Sphaerodactylus* spp. is found ancestral to *S. klauberi*. The other clade (Fig. S3; bottom) is composed of two subgroups. The first groups two species from Puerto Rico (*S. roosevelti* and *S. macrolepis*) and the other three species from Hispaniola (*S. clenchi, S. difficilis,* and *S. savagei*). There is a well-supported separation between the subgroups representing each of the two islands (98/92 for the Puerto Rico subgroup and 100/100 for the Hispaniola subgroup).

**Figure 3:**
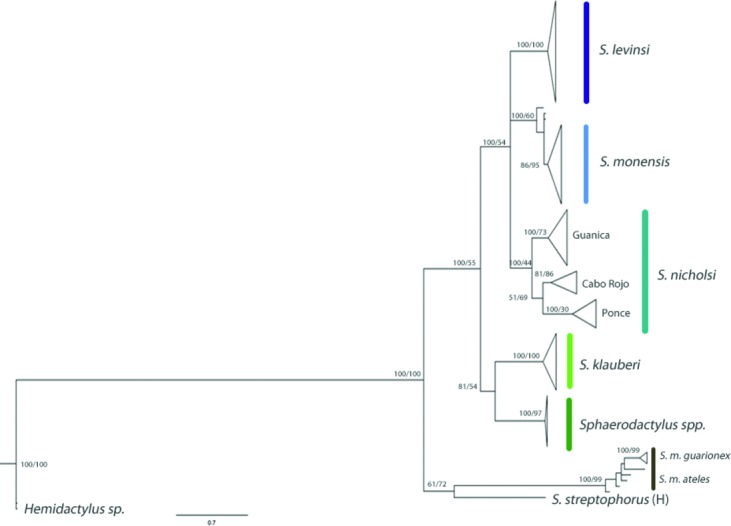
Phylogenetic tree constructed using the concatenated 16S and 12S rRNA sequences of *Sphaerodactylus* species from Puerto Rico, Mona, Desecheo and Hispaniola. Maximum likelihood and Bayesian inference algorithms produced identical topologies. All bootstrap values are shown for Bayesian inference and maximum likelihood trees in that order. Open triangles represent clusters of repeated haplotypes from the same species.

The 12S and 16S rRNA trees agree, as the median-joining networks, that the species in the Mona Passage are more closely related to the species from Puerto Rico than to the species from Hispaniola. They also agree in that *S. nicholsi* is the only species that forms a monophyletic group with the Mona Passage species. However, the 16S rRNA tree has two major differences with the 12S rRNA tree. First, the Mona Passage species form a monophyletic group in the 16S rRNA tree, but in the 12S rRNA tree, *S. nicholsi* becomes a sister species of *S. levinsi*. Second, *Sphaerodactylus* spp. is a sister species of *S. klauberi* in the 16S rRNA tree but is ancestral to *S. klauberi* in the 12S rRNA tree. A tree constructed with the concatenated sequences presents a trifurcation between *S. nicholsi*, *S. monensis*, and *S. levinsi*, and agrees with the 16S rRNA tree in the monophyletic origin of *Sphaerodactylus* spp. and *S. klauberi* (Fig. 3).

### Divergence time estimates

**Figure 4:**
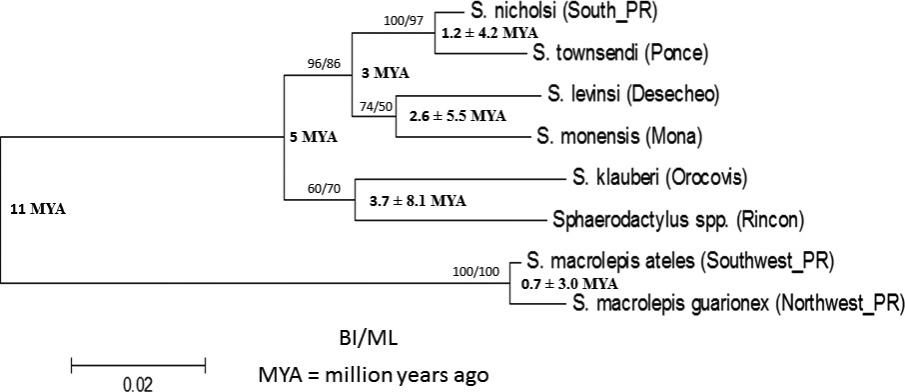
Phylogenetic tree constructed using the 16S and 12S rRNA consensus concatenated sequences of *Sphaerodactylus* from Puerto Rico, Mona and Desecheo. Bayesian inference and maximum likelihood algorithms showed identical topologies; only one consensus tree is shown with all bootstrap values. Bootstrap values for the Bayesian inference and maximum likelihood, algorithms are shown in that order. Mean divergence times in millions of years with standard deviations were calculated using a Bayesian relaxed clock. Time estimates were calculated independently of this phylogenetic tree, since the tree is not in a clock-like fashion. The regions of Puerto Rico where the samples were collected are shown in parentheses.

To estimate time of species divergence based on the full set of data from this study, we constructed a phylogenetic tree using the 16S and 12S rRNA concatenated consensus haplotype of each species of *Sphaerodactylus* sampled (Fig. 4). Pairs of consensus concatenated sequences were tested, and a mean divergence time estimate was calculated (Table 2). *S. macrolepis* forms the most ancestral divergence dating to around 11 Ma. The next earliest split separating the *S. klauberi* and *Sphaerodactylus* spp. pair from *S. nicholsi* and the species from the Mona Passage occurred around 5 Ma. Later is the split between *S. klauberi* and *Sphaerodactylus* spp., which occurred 3.7 Ma, much before the split between *S. macrolepis ateles* and *S. macrolepis guarionex* that occurred 0.7 Ma. *S. nicholsi* and the Mona Passage species diverged approximately 3 Ma, and at about 2.6 Ma, *S. monensis* and *S. levinsi* split into two different species. According to Coyne and Orr (1989, 1997, 1998), it takes an average of 1.5–3.5 Myrs of separation between sister species for them to acquire full reproductive isolation. This time interval is met by all of our taxon pairs except for the *S. macrolepis* subspecies. All the data together suggest that the dispersal event to the Mona Passage occurred around 3 Ma, with *S. monensis* diverging from *S. nicholsi* or a *proto-nicholsi* species, and *S. levinsi* diverging from a population in Mona Island around 2.6 Ma in a stepping-stone fashion (Fig. 1; bottom).

### Taxonomic placement of Sphaerodactylus spp

Ten of twelve *Sphaerodactylus* samples collected in Rincón could not be matched morphologically to any of the currently described species. The ten individuals shared the same 12S and 16S rRNA haplotypes, distinct from those of the other dwarf geckos in Puerto Rico. In every analysis, *Sphaerodactylus* spp. distinctly formed its own monophyletic group, reasonably separating itself from its closest relative, *S. klauberi*. In fact, there are more differences between the two of them (consensus sequences diverged 5.6% from each other) than between the two known *S. macrolepis* subspecies (consensus sequences diverged 1.2% from each other) and about the same amount of differences that can be found between any two established species of *Sphaerodactylus* (7.3% divergence between *S. nicholsi* and *S. klauberi*). This gives us grounds to state that *Sphaerodactylus* spp. is possibly a new, previously undescribed species that will be officially described using a combination of morphological and molecular methods in a separate publication (in preparation).

## Discussion

### Patterns of *Sphaerodactylus* dispersal in the Mona Passage

Isolated oceanic islands such as Mona and Desecheo are interesting to biologists that study colonization, succession, and other evolutionary and ecological processes. Biologic diversity on these islands is mostly a result of dispersion from either a continent or another island in proximity. In the case of Mona and Desecheo, the closest lands are the island of Hispaniola to the west and Puerto Rico to the east, both potential donors of migrants. In this study, we used molecular data to investigate the colonization routes of two endemic vertebrate species found on these islands. Our analysis suggests that the genus *Sphaerodactylus* that colonized Mona and Desecheo originally came from the southwestern part of the island of Puerto Rico (Fig. 1). This pattern has been previously observed in other organisms of the Mona Passage islands that can be dispersed by wind, considering that the winds that come from Africa usually blow from southeast to northwest (Griffin et al. 2001), such as in certain vascular plants (Woodbury et al. 1977) and butterflies (Smith et al. 1994).

Although overwater dispersion is a less likely event than dispersal by wind, it has been demonstrated to occur in the Caribbean, and Hedges et al. (1992) proposed that overwater dispersion is the primary mechanism of colonization in the West Indies. We propose that the ancestor species accidentally traveled on flotsams formed in southwestern Puerto Rico (Guánica region) to Mona where similar climate conditions facilitated colonization. Once there, it populated the island and diverged. A subset of this original Mona population then dispersed to Desecheo where they thrived in the valleys. In summary, a stepping-stone process occurred, where dwarf geckos first colonized one island and evolved there for a while, and later, a subset of this population colonized the next island. Our results are congruent with earlier studies focusing on dispersal patterns of other vertebrate species, like *Anolis*, that also have colonized Mona and Desecheo from southwestern Puerto Rico (Rodríguez-Robles et al. 2007). This recurrent biogeographical pattern can be explained by existing water currents that typically run from southeast to northwest (Griffin et al. 2001), as well as by the similarity of the climate between Mona Island and the southwestern part of Puerto Rico that share the same ecological life zone: a subtropical dry forest (Ewel and Whitmore 1973). Losos et al. (1997) suggested that colonizing populations adapt rapidly, in their case 10–14 years, to new environmental conditions. When the colonized environment is similar to the environment of origin, colonization is facilitated as the organism is already somewhat adapted to the climate and vegetation it needs to survive.

All our 12S rRNA, 16S rRNA, and concatenated sequence networks (Figs. S1 and S2) and phylogenetic trees (Fig. 3, S3 and S4) clearly show that *S. nicholsi*, from southwestern Puerto Rico, is more closely related to the Mona Passage species than any other species in Puerto Rico or Hispaniola. Furthermore, we have circumscribed the most likely area from which the flotsam that gave rise to the Mona Passage species originated to Guánica. Figure 1 dramatizes this conclusion, first because all *S. nicholsi* haplotypes cluster according to location, and second because the Guánica cluster is not found closer to the Mona Passage species simply through a closely related shared ancestor, but by actually standing in the network right between *S. monensis* and *S. nicholsi* from Cabo Rojo, the next closest taxon. This leaves few doubts that the *S. nicholsi* population from Guánica is more closely related to *S. monensis* than the population from Cabo Rojo, at least by the mtDNA criterium. The amount of mutations accumulated between *S. levinsi* and *S. monensis* may be a reflection of the founder effect that occurred after the colonization of Desecheo: the effective population size of the colonizing species decreases, reducing selective pressure over new mutations and thus increasing the chances for new mutations to accumulate, creating new haplotypes, and increasing the genetic divergence between the species (Nei et al. 1975). The same pattern is observed between *S. nicholsi* and *S. monensis*, which is consistent with the stepping-stone colonization model that we propose.

Figures 1 and 2 together also show that the hypothetical ancestor most closely related to *S. levinsi* is only one mutational step away from a *S. monensis* haplotype found in El Faro and two mutational steps from the putative founder *S. monensis* haplotype. This suggests that an area near El Faro, at the eastern tip of Mona Island, may have been the origin of the flotsam that gave rise to *S. levinsi* on Desecheo.

The stepwise model is also consistent with the fact that the *S. levinsi* has lower haplotype diversity than *S. monensis*. This suggests that the Desecheo gecko may indeed be historically younger, so fewer mutations could have accumulated there. Nonetheless, alternate explanations can be invoked. Predation from monkeys in Desecheo, absent in Mona, could have caused a recent population bottleneck. In addition, because Desecheo Island is much smaller than Mona, the populations there could also have smaller effective population sizes and be more affected by genetic drift, leading to reduced genetic diversity. Finally, a sampling bias might have taken place because most of the Desecheo samples were collected in the same locality. However, we believe the last two alternatives to be unlikely because the sample set of every single collecting site on Mona Island had more variation than the Desecheo sample (Figs. 1 and 2).

It is important to note that our conclusions on the stepping-stone fashion colonization of the Mona Passage islands and on *S. klauberi* and *Sphaerodactylus* spp. forming a monophyletic group cannot be reached by analyzing only one of the rRNA genes. We solve this problem by concatenating the sequences. Concatenation is legitimate because of the nonrecombining nature of mtDNA, which thus segregates as a single locus. On concatenation, the phylogenetic tree strongly supports a root between *S. macrolepis* on one side, and *Sphaerodactylus* spp.*, S. klauberi, S. nicholsi,* and the Mona Passage species on the other. This allows us to conclude that the root in the networks does not lie within the *S. nicholsi*–*S. monensis*–*S. levinsi* trio that appear as a closely related group in all networks. Networks are phylogenetic representations that map genetic relationships between closely related organisms without a specific root or known ancestor. Between more distantly related species, homoplasies occur often enough to obscure these genetic relationships, and this explains the inconsistencies in the networks on how more distantly related species are connected to this trio of more closely related species. By placing the root outside the trio, we can reach the conclusion on the stepwise fashion colonization of the Mona Passage from *S. nicholsi* in Guánica (Figs. S2 and 1).

Although all networks, the 16S rRNA tree and the concatenated sequences tree place *Sphaerodactylus* spp. as the most closely related species to *S. klauberi*, the 12S rRNA tree places *Sphaerodactylus* spp. with a deeper root. This disagreement may be due to stochastic processes aggravated by the small effective population size of mtDNA and by the severely restricted gene flow that is becoming apparent between dwarf gecko populations. Dwarf geckoes are susceptible to thermal stress and dehydration because they are xerophytic forest reptiles of small body size, relatively large surface area/volume ratio, and diurnal activity. These conditions may restrict their mobility and provoke limited gene flow between populations, thus increasing genetic drift and the chances that different mtDNA segments evolve differently.

### Evolution of *Sphaerodactylus* in Puerto Rico

Hass (1991) proposed Hispaniola as the center of dispersion for *Sphaerodactylus* during the Proto-Antilles period because of the high species diversity of this group on the island. As the Greater Antilles were joined together, the *Sphaerodactylus* species of the time may have colonized the entire land mass called the Proto-Antilles, consisting of the combined territories of Cuba, Hispaniola, and Puerto Rico. Then, as the islands separated by tectonic plate movement 15–20 Ma, the geckos also separated and diverged vicariantly into a number of different island species. Dated amber fossils show that sphaerodactylids existed in Hispaniola during this time period (Iturralde-Vinent and MacPhee 1996; Daza and Bauer 2012; Daza et al. 2012). Our phylogenetic trees present at least two Hispaniolan clades (Fig. S4) and possibly another nesting with *S. macrolepis* and *S. roosevelti* of Puerto Rico (Fig. S3). However, all Puerto Rican species are assorted into only two clades: *S. macrolepis* and *S. roosevelti* form a clade apart from *S. nicholsi* and the other species in Puerto Rico (Figs. 3, S3 and S4). The separation of the *S. macrolepis*–*S. roosevelti* lineage from the *S. nicholsi* lineage is estimated to have occurred 11 Ma in the Miocene (Fig. 4; Table 2). This date coincides with the vicariance event proposed by Gamble et al. (2008a) between *S. ocoae* of Hispaniola and *S. roosevelti* of Puerto Rico, and it would thus be important to study the phylogenetic relationship of the latter two species with *S. nicholsi* under the expectation that *S. ocoae* would not show a significantly closer relationship to *S. roosevelti* than to *S. nicholsi*. Our results suggest that the three Hispaniolan species nesting with *S. macrolepis* (Fig. S3) could have diverged from a *proto-macrolepis* ancestor only after the *proto*-macrolepis–*S. nicholsi* split. This would suggest that a cross-Mona Passage colonization event occurred after 11 Ma, either to give rise to *S. macrolepis* in Puerto Rico from an ancestor migrating from Hispaniola or to give rise to Hispaniolan species from a *proto-macrolepis* ancestor in Puerto Rico.

Our results do not support the morphology-based conclusions of Thomas and Schwartz (1966) that place *S. macrolepis* as the most ancestral species of the ones studied. In addition, they do not support their conclusion that *S. monensis* evolved from a *proto-macrolepis* ancestor. Our results indicate that *S. monensis* evolved much later (only 3–2 Ma as opposed to *S. macrolepis* that diverged around 11 Ma) from *S. nicholsi*.

While *S. townsendi* and *S. nicholsi* have been considered distinct species, we did not find support for this separation. The two samples of *S. townsendi* that were collected in Ponce always clustered with *S. nicholsi* both in the phylogenetic trees and in the median networks (Figs. 1, 4, S1 and S2) did not form their own monophyletic group, and even shared a haplotype with *S. nicholsi*. The presence of *S. townsendi* in the concatenated consensus sequences tree (Fig. 4) resolved the trifurcation observed in the concatenated sequences tree (Fig. 3). However, at this point, we cannot rule out other causes for this lack of separation between these two species such as the emergence of natural hybrids because natural hybrids between *S. nicholsi* and *S. townsendi* have been previously reported by Murphy et al. (1984). Considering that we used mtDNA, inherited only from the mother, it is possible that the specimens we examined were either the first or further generations of hybrids from *S. townsendi* fathers and *S. nicholsi* mothers. Under this scenario, the molecular phylogenetic tools we used would classify such individuals as *S. nicholsi*, independently of their morphological traits.

## Conclusions

The natural history of the genus *Sphaerodactylus* in Puerto Rico and the Mona Passage could have followed this sequence of events: approximately 11 Ma a split between two major lineages of different species in Puerto Rico occurred. One lineage gave rise to *S. roosevelti* and the nine subspecies of *S. macrolepis*. The other lineage gave rise to *S. klauberi* in higher altitudes and *S. nicholsi* in the dry forest areas. A subset of the *S. nicholsi* population from the area around Guánica traveled on a flotsam, aided by the ocean currents, to Mona Island, were it found a habitat similar to its own and successfully colonized the island around 3 Ma. Then, after another dispersion event, colonists from around El Faro on Mona Island traveled to Desecheo, established a new population there, and eventually diverged to the new species known today as *S. levinsi*.

## Data Accessibility

DNA sequences: GenBank accessions KC840429-KC840514 (12S rRNA), KC840515-KC840608 (16S rRNA).

Phylogenetic data and DNA sequence alignments: TreeBASE Reviewer access URL: http://purl.org/phylo/treebase/phylows/study/TB2:S14078?x-access-code=deabfe277cdf37eb616568f9e892e769&format=html.

## Supporting Information

**Figure S1**. Median joining network of 16S rRNA (Panel A) and 12S rRNA (Panel B) sequences of *Sphaerodactylus* species from Hispaniola, Puerto Rico, Mona and Desecheo. Colored circles indicate different haplotypes, the size of each circle is equivalent to its haplotype frequency. Hypothetical ancestors are represented by red diamonds. Distances exceeding three mutations separating haplotypes and hypothetical ancestors are indicated in the corresponding branches.

**Figure S2**. Median joining network of 16S and 12S rRNA concatenated sequences of *Sphaerodactylus* species from Puerto Rico, Mona and Desecheo. Colored circles indicate different haplotypes; the size of each circle is proportional to its haplotype frequency. Hypothetical ancestors are represented by red diamonds. Distances exceeding three mutations separating haplotypes and hypothetical ancestors are indicated in the corresponding branches.

**Figure S3**. Phylogenetic tree for the 12S rRNA sequences of *Sphaerodactylus* species from Puerto Rico, Mona, Desecheo, Hispaniola, and various Lesser Antilles. Taxa without identification of the specific island of origin are from Puerto Rico, Mona or Desecheo. *Hemidactylus spp*. collected in Puerto Rico were used as outgroups. All bootstrap values are shown for Bayesian inference and maximum likelihood trees in that order. Open triangles represent clusters of repeated haplotypes from the same species. LA = Lesser Antilles, H = Hispaniola

**Figure S4.** Phylogenetic tree for the 16S rRNA sequences of *Sphaerodactylus* species from Puerto Rico, Mona, Desecheo, Hispaniola (H), Cuba (C), Jamaica (J), Florida (F) and the Lesser Antilles (LA). Taxa without identification of the specific island of origin are from Puerto Rico, Mona or Desecheo. *Hemidactylus spp*. collected in Puerto Rico where used as outgroups. Samples downloaded from NCBI are properly indicated with the anachronism (Table S1). All bootstrap values are shown in the same tree order: maximum likelihood, maximum parsimony and neighbor joining. Open triangles are clusters of repeated haplotypes from the same species.

**Table S1**. Sequence data downloaded from NCBI Genbank with reference and accession number.
