# Supplementary material for: Colonization of islands in the Mona Passage by endemic dwarf geckoes (genus Sphaerodactylus) reconstructed with mitochondrial phylogeny
Source: Ecol Evol. 2013 Oct 16;3(13):4488–500. doi: 10.1002/ece3.770 (PMC3856748; doi:10.1002/ece3.770)
Supplement: Supplementary file 1 [file ece30003-4488-SD1.docx]

**Colonization of Islands in the Mona Passage by Endemic Dwarf Geckoes (genus *Sphaerodactylus*) Reconstructed with Mitochondrial Phylogeny**

Díaz-Lameiro et al.

**Supplementary Materials**

**Supplemental Information 1, Figure S1**. Median joining network of 16S rRNA (Panel A) and 12S rRNA (Panel B) sequences of *Sphaerodactylus* species from Hispaniola, Puerto Rico, Mona and Desecheo. Colored circles indicate different haplotypes, the size of each circle is equivalent to its haplotype frequency. Hypothetical ancestors are represented by red diamonds. Distances exceeding three mutations separating haplotypes and hypothetical ancestors are indicated in the
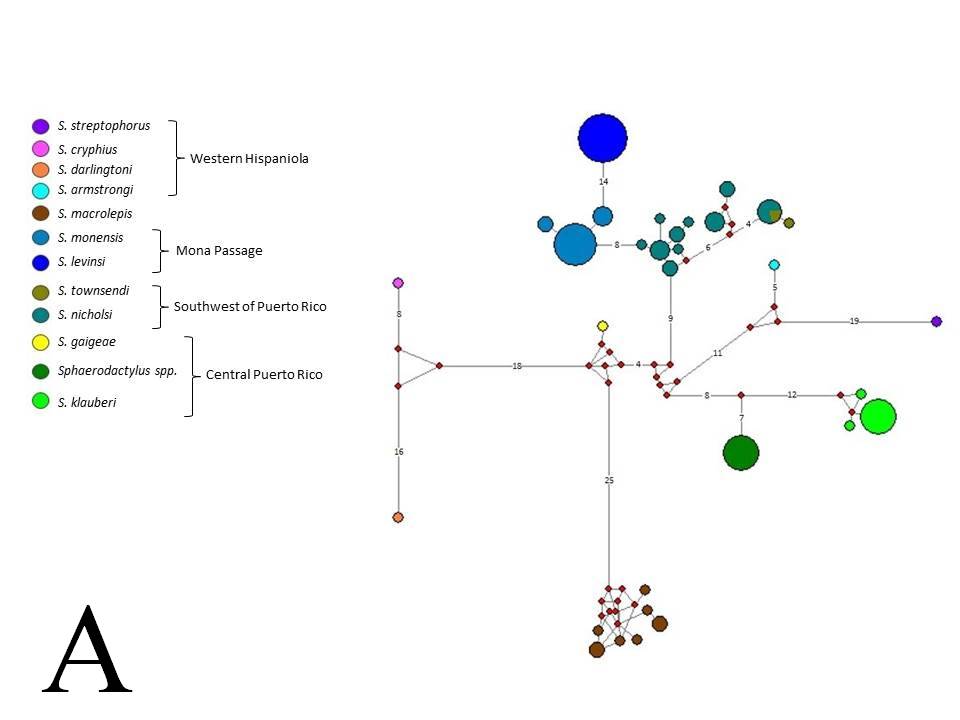
corresponding branches.


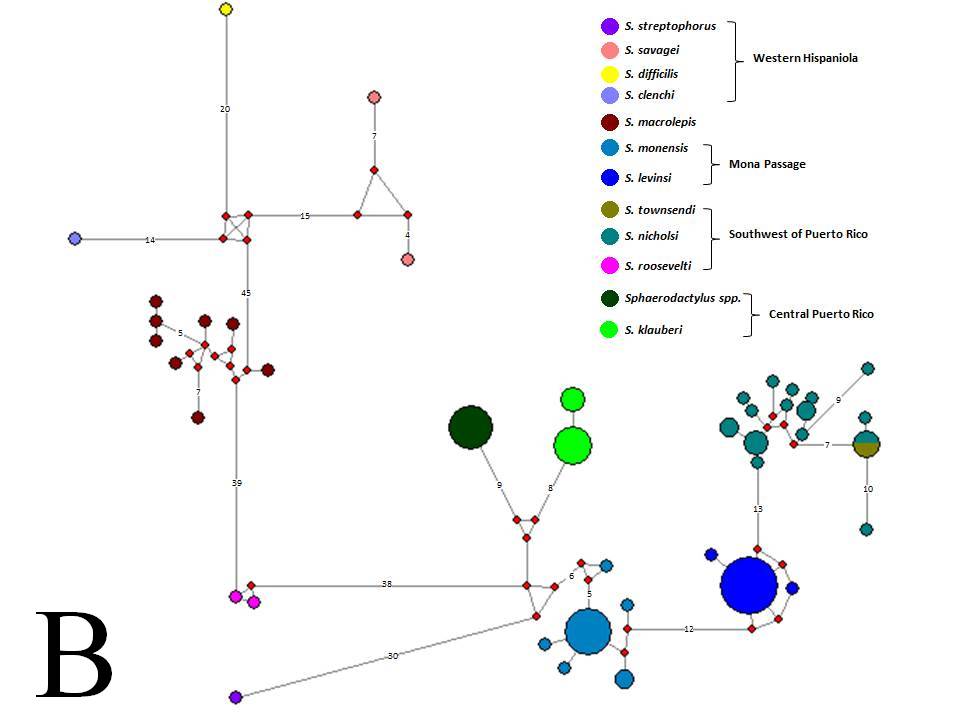


**Supplemental Information 2, Figure S2**. Median joining network of 16S and 12S rRNA concatenated sequences of *Sphaerodactylus* species from Puerto Rico, Mona and Desecheo. Colored circles indicate different haplotypes; the size of each circle is proportional to its haplotype frequency. Hypothetical ancestors are represented by red diamonds. Distances exceeding three mutations separating haplotypes and hypothetical ancestors are indicated in the
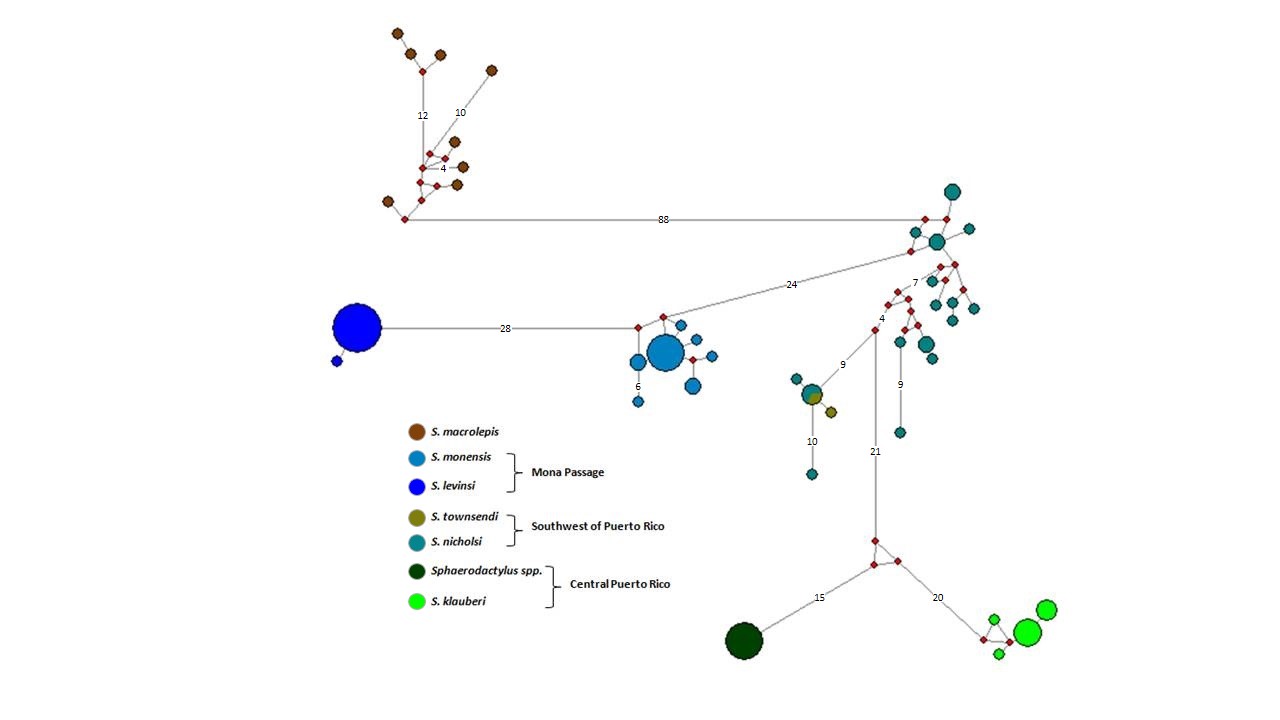
corresponding branches.

**Supplemental Information 3, Figure S3** Phylogenetic tree for the 12S rRNA sequences of *Sphaerodactylus* species from Puerto Rico, Mona, Desecheo, Hispaniola, and various Lesser Antilles. Taxa without identification of the specific island of origin are from Puerto Rico, Mona or Desecheo. *Hemidactylus spp*. collected in Puerto Rico were used as outgroups. All bootstrap values are shown for Bayesian inference and maximum likelihood trees in that order. Open triangles represent clusters of repeated haplotypes from the same species. LA = Lesser Antilles, H = Hispaniola


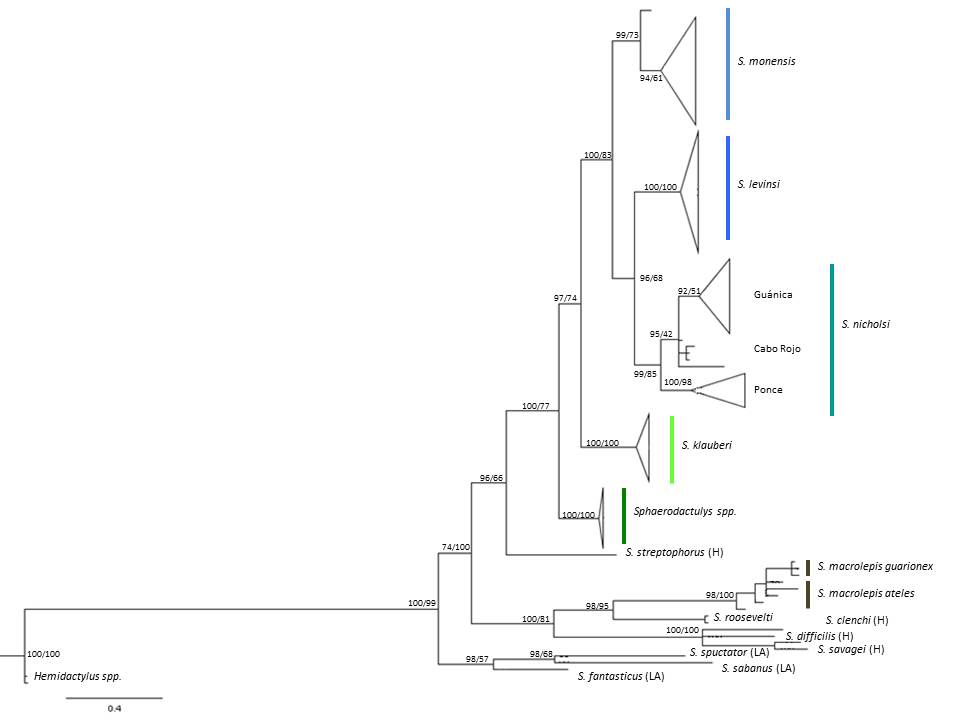


**Supporting Information 4, Figure S4** Phylogenetic tree for the 16S rRNA sequences of *Sphaerodactylus* species from Puerto Rico, Mona, Desecheo, Hispaniola (H), Cuba (C), Jamaica (J), Florida (F) and the Lesser Antilles (LA). Taxa without identification of the specific island of origin are from Puerto Rico, Mona or Desecheo. *Hemidactylus spp*. collected in Puerto Rico where used as outgroups. Samples downloaded from NCBI are properly indicated with the anachronism (Table S1). All bootstrap values are shown in the same tree order: maximum likelihood, maximum parsimony and neighbor joining. Open triangles are clusters of repeated
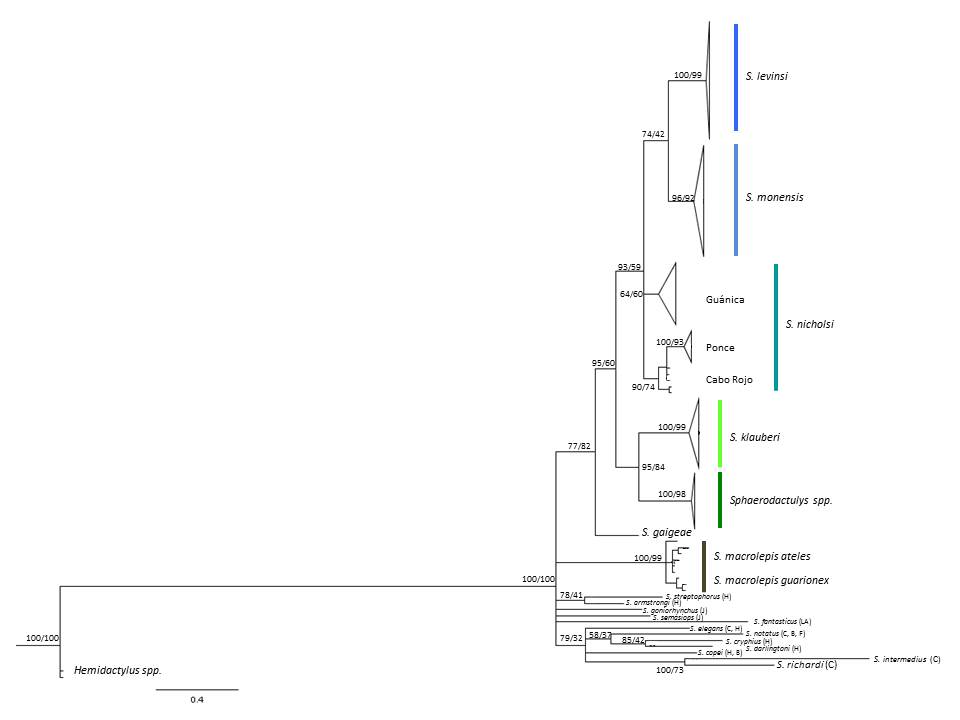
haplotypes from the same species.

**Supplemental Information 5, Table S1**. Sequence data downloaded from NCBI Genbank with reference and accession number.

| **Species** | **Accession Code** | **Reference** | **Fragment** |
| --- | --- | --- | --- |
| *S. armstrongi* | X86038 | Hass 1992 | 16S rRNA |
| *S. copei* | X86062 | Hass 1992 | 16S rRNA |
| *S. cryphius* | X86039 | Hass 1992 | 16S rRNA |
| *S. darlingtoni* | X86040 | Hass 1992 | 16S rRNA |
| *S. elegans* | X86048 | Hass 1992 | 16S rRNA |
| *S. fantasticus* | X86058 | Hass 1992 | 16S rRNA |
| *S. gaigeae* | X86042 | Hass 1992 | 16S rRNA |
| *S. goniorhynchus* | X86041 | Hass 1992 | 16S rRNA |
| *S. intermedius* | X86050 | Hass 1992 | 16S rRNA |
| *S. notatus* | X86061 | Hass 1992 | 16S rRNA |
| *S. richardi* | X86054 | Hass 1992 | 16S rRNA |
| *S. semasiops* | X86045 | Hass 1992 | 16S rRNA |
| *S. fantasticus* | EU191670 | Thorpe et al. 2008 | 12S rRNA |
| *S. roosevelti* | DQ852713 | Feng et al. 2007 | 12S rRNA |
| *S. sabanus* | EU191623 | Thorpe et al. 2008 | 12S rRNA |
| *S. sputator* | EU191624 | Thorpe et al. 2008 | 12S rRNA |
