# Supplementary material for: Colonization of islands in the Mona Passage by endemic dwarf geckoes (genus Sphaerodactylus) reconstructed with mitochondrial phylogeny
Source: Ecol Evol. 2013 Oct 16;3(13):4488–500. doi: 10.1002/ece3.770 (PMC3856748; doi:10.1002/ece3.770)
Supplement: Supplementary file 2 [file ece30003-4488-SD2.pdf]

Colonization of Islands in the Mona Passage by Endemic Dwarf Geckoes (genus *Sphaerodactylus*) Reconstructed with Mitochondrial Phylogeny

Díaz-Lameiro et al.

Supplementary Materials

Supplemental Information 1, Figure S1. Median joining network of 16S rRNA (Panel A) and 12S rRNA (Panel B) sequences of *Sphaerodactylus* species from Hispaniola, Puerto Rico, Mona and Desecheo. Colored circles indicate different haplotypes, the size of each circle is equivalent to its haplotype frequency. Hypothetical ancestors are represented by red diamonds. Distances exceeding three mutations separating haplotypes and hypothetical ancestors are indicated in the corresponding branches.

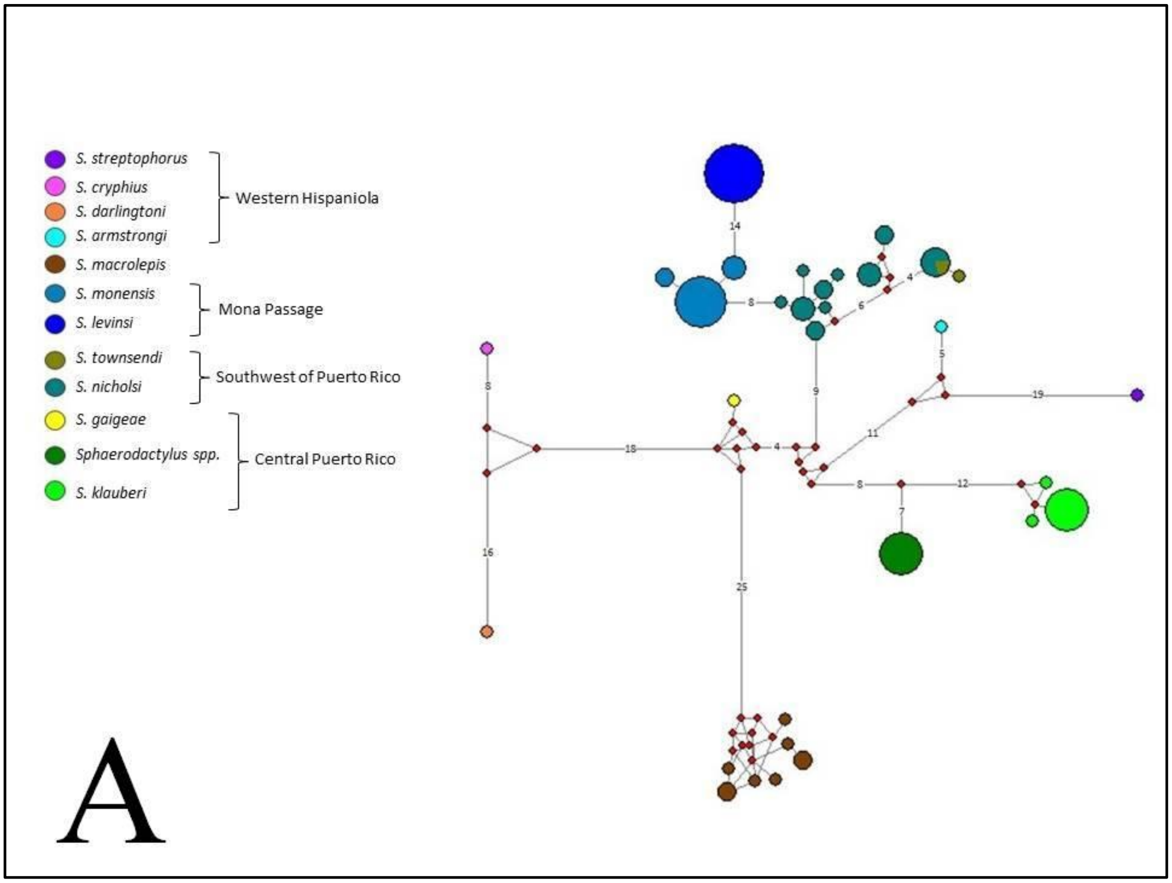

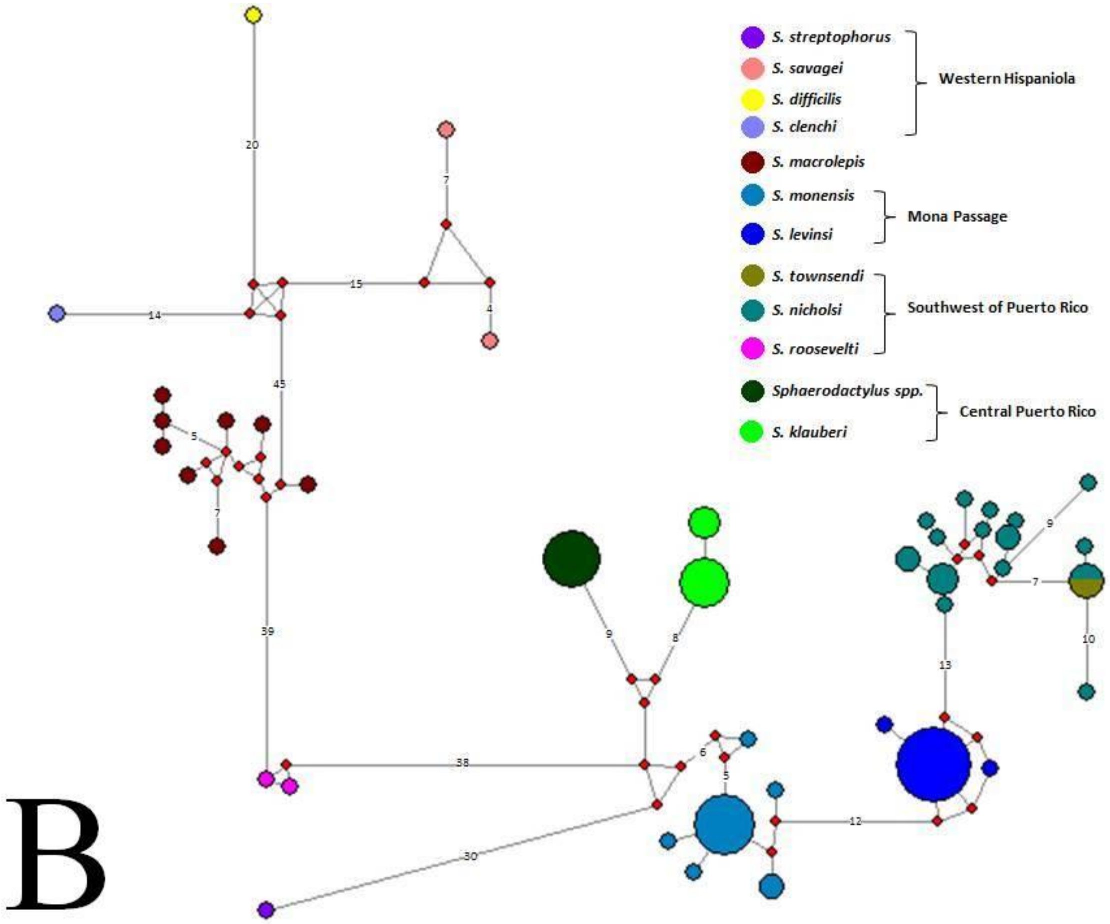
S. streptophorus
S. savagei
S. difficilis
S. clenchi
Western Hispaniola
S. macrolepis
S. monensis
S. levinsi
Mona Passage
S. townsendi
S. nicholsi
S. roosevelti
Southwest of Puerto Rico
Sphaerodactylus spp.
S. klauberi
Central Puerto Rico
B

Supplemental Information 2, Figure S2. Median joining network of 16S and 12S rRNA concatenated sequences of *Sphaerodactylus* species from Puerto Rico, Mona and Desecheo. Colored circles indicate different haplotypes; the size of each circle is proportional to its haplotype frequency. Hypothetical ancestors are represented by red diamonds. Distances exceeding three mutations separating haplotypes and hypothetical ancestors are indicated in the corresponding branches.

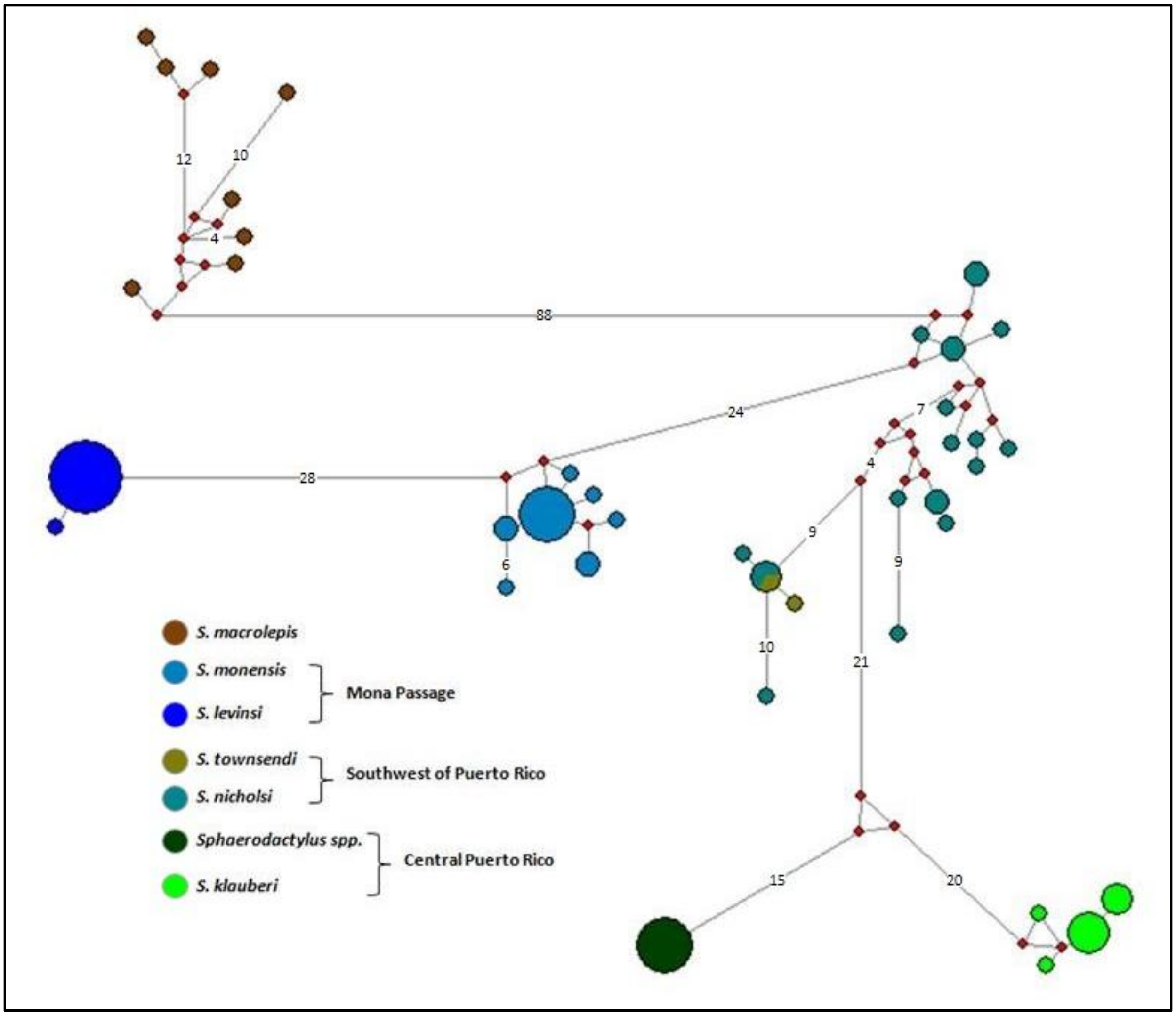

Supplemental Information 3, Figure S3 Phylogenetic tree for the 12S rRNA sequences of *Sphaerodactylus* species from Puerto Rico, Mona, Desecheo, Hispaniola, and various Lesser Antilles. Taxa without identification of the specific island of origin are from Puerto Rico, Mona or Desecheo. *Hemidactylus spp*. collected in Puerto Rico were used as outgroups. All bootstrap values are shown for Bayesian inference and maximum likelihood trees in that order. Open triangles represent clusters of repeated haplotypes from the same species. LA = Lesser Antilles, H = Hispaniola.

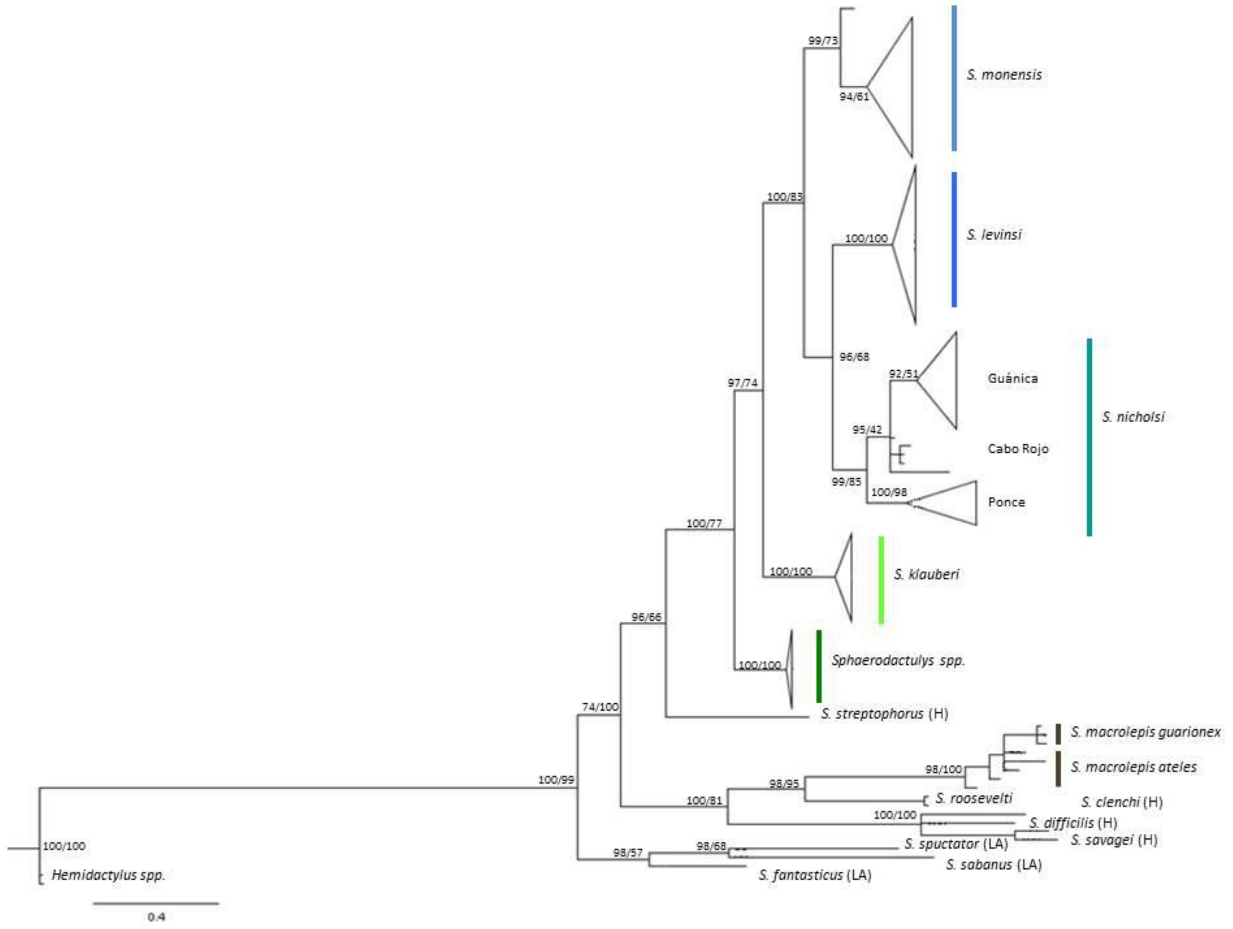

Supporting Information 4, Figure S4 Phylogenetic tree for the 16S rRNA sequences of *Sphaerodactylus* species from Puerto Rico, Mona, Desecheo, Hispaniola (H), Cuba (C), Jamaica (J), Florida (F) and the Lesser Antilles (LA). Taxa without identification of the specific island of origin are from Puerto Rico, Mona or Desecheo. *Hemidactylus spp*. collected in Puerto Rico where used as outgroups. Samples downloaded from NCBI are properly indicated with the anachronism (Table S1). All bootstrap values are shown in the same tree order: maximum likelihood, maximum parsimony and neighbor joining. Open triangles are clusters of repeated haplotypes from the same species.

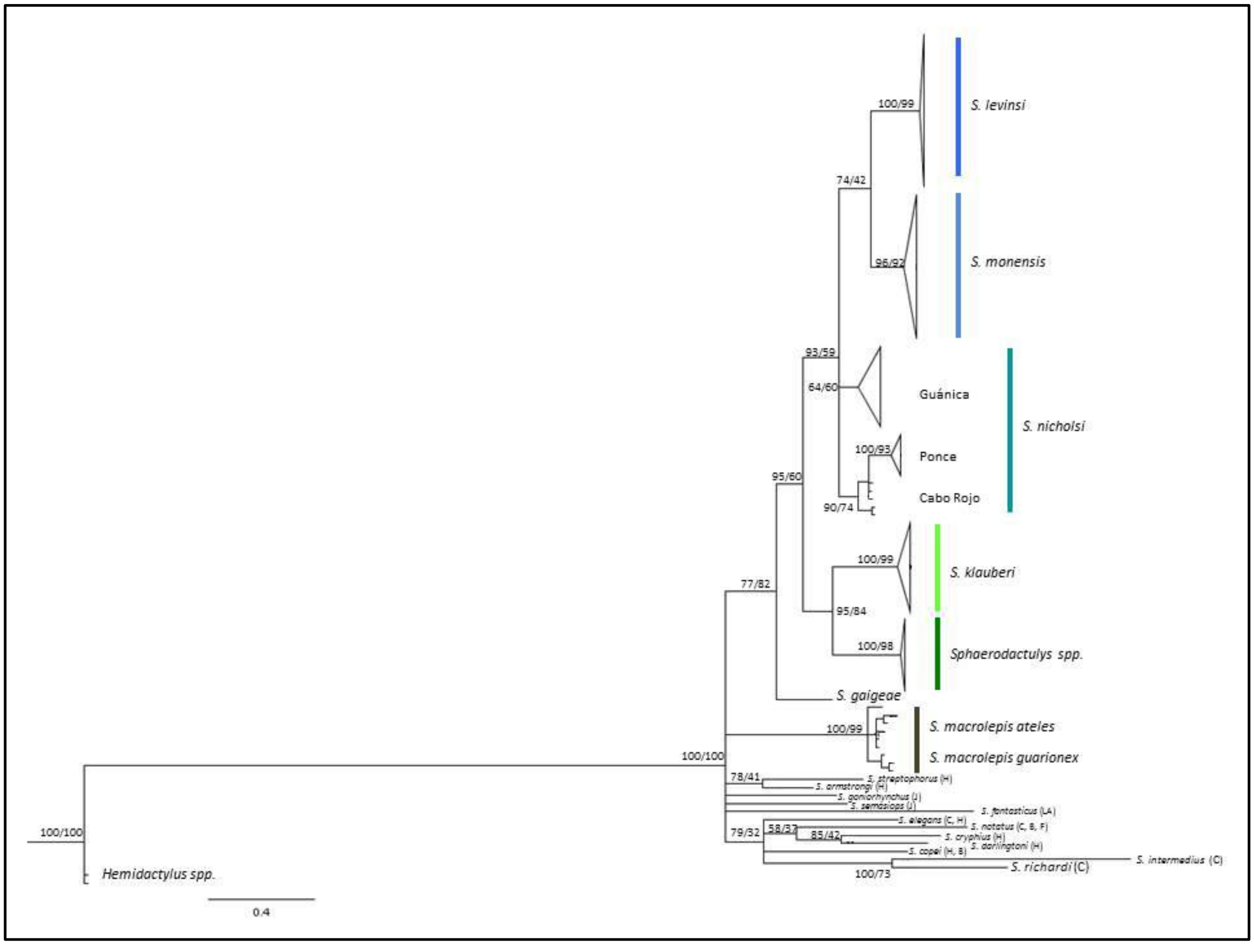

Supplemental Information 5, Table S1. Sequence data downloaded from NCBI Genbank with reference and accession number.

Species	Accession Code	Reference	Fragment
S. armstrongi	X86038	Hass 1996	16S rRNA
S. copei	X86062	Hass 1996	16S rRNA
S. cryphius	X86039	Hass 1996	16S rRNA
S. darlingtoni	X86040	Hass 1996	16S rRNA
S. elegans	X86048	Hass 1996	16S rRNA
S. fantasticus	X86058	Hass 1996	16S rRNA
S. gaigeae	X86042	Hass 1996	16S rRNA
S. goniorhynchus	X86041	Hass 1996	16S rRNA
S. intermedius	X86050	Hass 1996	16S rRNA
S. notatus	X86061	Hass 1996	16S rRNA
S. richardi	X86054	Hass 1996	16S rRNA
S. semasiops	X86045	Hass 1996	16S rRNA
S. fantasticus	EU191670	Thorpe et al. 2008	12S rRNA
S. roosevelti	DQ852713	Feng et al. 2007	12S rRNA
S. sabanus	EU191623	Thorpe et al. 2008	12S rRNA
S. sputator	EU191624	Thorpe et al. 2008	12S rRNA

References

Feng J, Han D, Bauer AM, Zhou K (2007) Interrelationships among gekkonid geckos inferred from mitochondrial and nuclear gene sequences. *Zoological Science*, **24**, 656-665.

Hass CA (1996) Relationships among West Indian geckos of the genus *Sphaerodactylus*: a preliminary analysis of mitochondrial 16S ribosomal RNA sequences. In: Contributions to West Indian Herpetology: A Tribute to Albert Schwartz (eds Powell R, Henderson R), pp.175-194. Society for the Study of Amphibians and Reptiles, Ithaca, NY. Contributions to Herpetology, volume 12.

Thorpe RS, Jones AG, Malhotra A, Surget-Groba Y (2008) Adaptive radiation in Lesser Antillean lizards: molecular phylogenetics and species recognition in the Lesser Antillean dwarf gecko complex *Sphaerodactylus fantasticus*. *Molecular Ecology*, **17**, 1489-1504.
