# Supplementary figures and images for: Colonization of islands in the Mona Passage by endemic dwarf geckoes (genus Sphaerodactylus) reconstructed with mitochondrial phylogeny
Source: Ecol Evol. 2013 Oct 16;3(13):4488–500. doi: 10.1002/ece3.770 (PMC3856748; doi:10.1002/ece3.770)

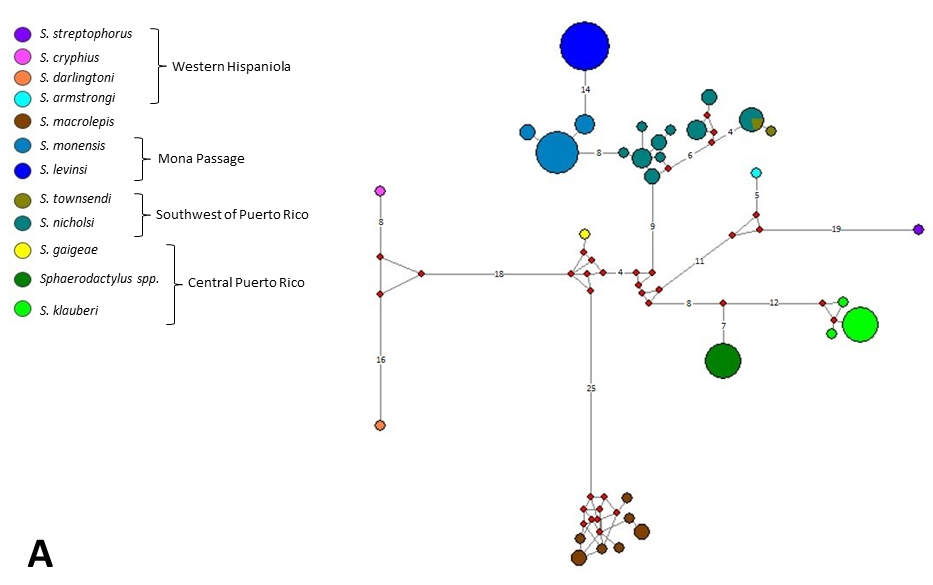

Supplement: Supplementary file 3 [file ece30003-4488-SD3.tif]

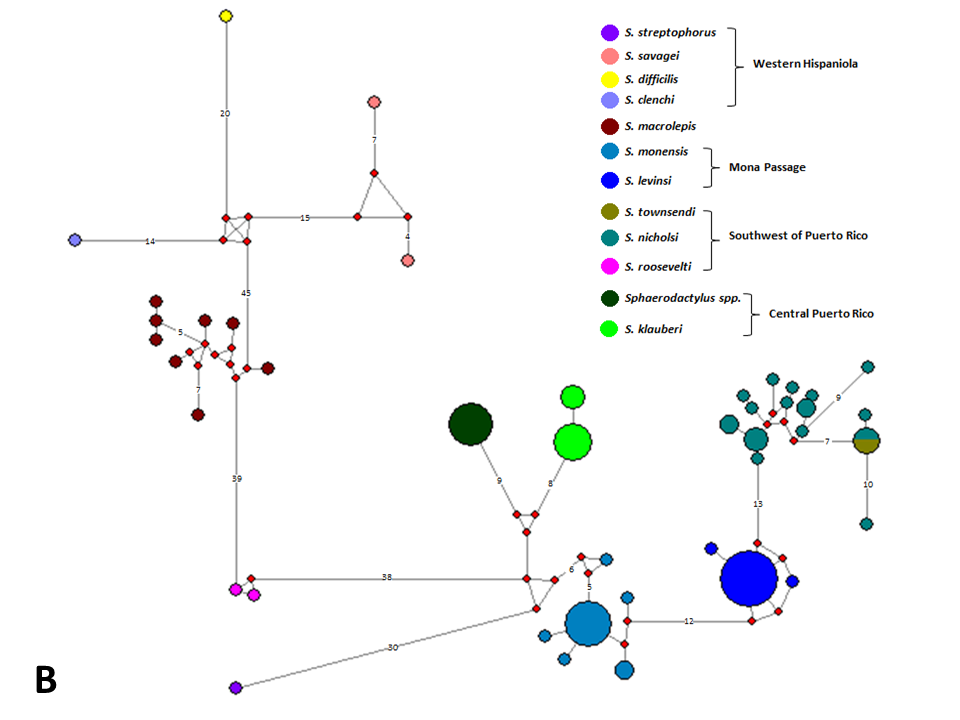

Supplement: Supplementary file 4 [file ece30003-4488-SD4.tif]

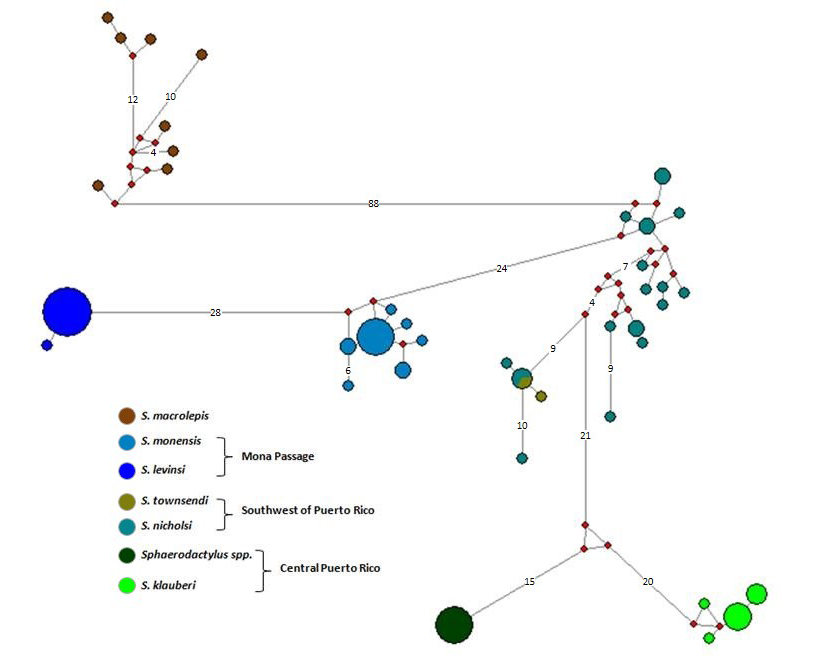

Supplement: Supplementary file 5 [file ece30003-4488-SD5.tif]

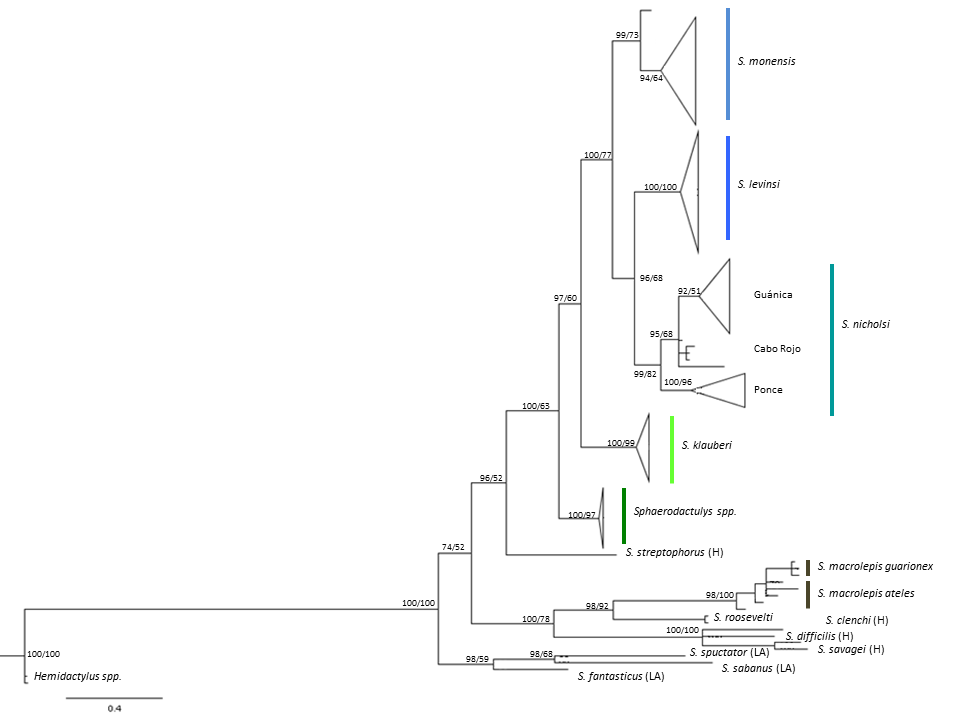

Supplement: Supplementary file 6 [file ece30003-4488-SD6.tif]

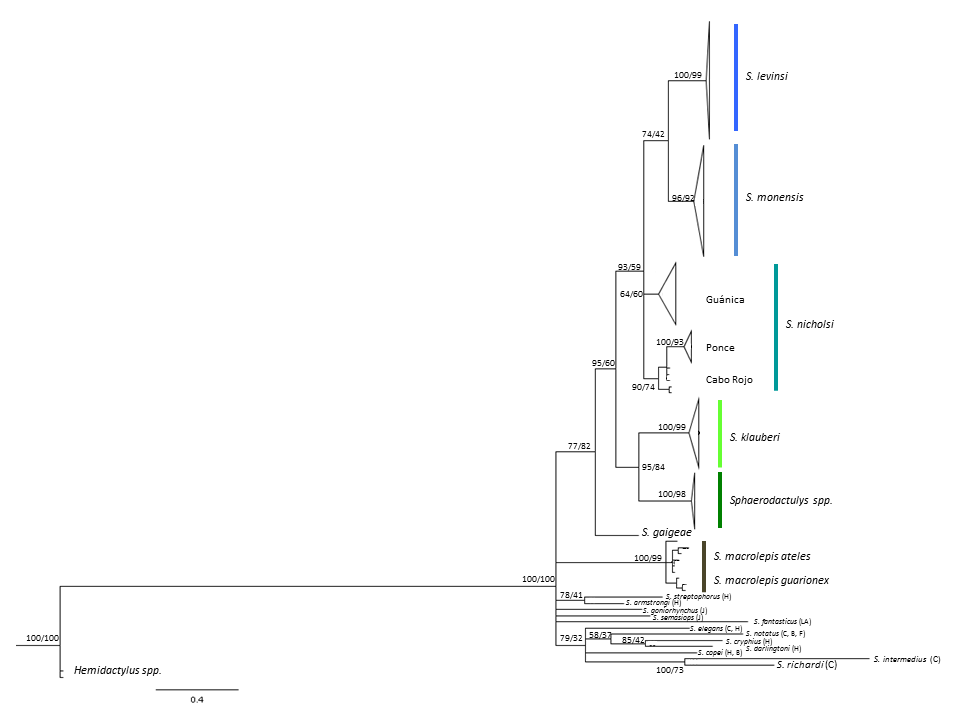

Supplement: Supplementary file 7 [file ece30003-4488-SD7.tif]
